# Systematic Analysis of Aberrant Biochemical Networks and Potential Drug Vulnerabilities Induced by Tumor Suppressor Loss in Malignant Pleural Mesothelioma

**DOI:** 10.3390/cancers12082310

**Published:** 2020-08-17

**Authors:** Haitang Yang, Duo Xu, Zhang Yang, Feng Yao, Heng Zhao, Ralph A. Schmid, Ren-Wang Peng

**Affiliations:** 1 Division of General Thoracic Surgery, Department of BioMedical Research (DBMR), Inselspital, Bern University Hospital, University of Bern, Murtenstrasse 50, CH3008 Bern, Switzerland; haitang.yang@dbmr.unibe.ch (H.Y.); duo.xu@dbmr.unibe.ch (D.X.); zhang.yang@dbmr.unibe.ch (Z.Y.); 2 Department of Thoracic Surgery, Shanghai Chest Hospital, Shanghai Jiao Tong University, Shanghai 200030, China; feng.yao@shchest.org (F.Y.); zh148@shchest.org (H.Z.)

**Keywords:** mesothelioma, tumor suppressor, targeted therapy, immunotherapy

## Abstract

*Background*: Malignant pleural mesothelioma (MPM) is driven by the inactivation of tumor suppressor genes (TSGs). An unmet need in the field is the translation of the genomic landscape into effective TSG-specific therapies. *Methods*: We correlated genomes against transcriptomes of patients’ MPM tumors, by weighted gene co-expression network analysis (WGCNA). The identified aberrant biochemical networks and potential drug targets induced by tumor suppressor loss were validated by integrative data analysis and functional interrogation. *Results*: CDKN2A/2B loss activates G2/M checkpoint and PI3K/AKT, prioritizing a co-targeting strategy for CDKN2A/2B-null MPM. CDKN2A deficiency significantly co-occurs with deletions of anti-viral type I interferon (IFN-I) genes and BAP1 mutations, that enriches the IFN-I signature, stratifying a unique subset, with deficient IFN-I, but proficient BAP1 for oncolytic viral immunotherapies. Aberrant p53 attenuates differentiation and SETD2 loss acquires the dependency on EGFRs, highlighting the potential of differentiation therapy and pan-EGFR inhibitors for these subpopulations, respectively. LATS2 deficiency is linked with dysregulated immunoregulation, suggesting a rationale for immune checkpoint blockade. Finally, multiple lines of evidence support Dasatinib as a promising therapeutic for LATS2-mutant MPM. *Conclusions*: Systematic identification of abnormal cellular processes and potential drug vulnerabilities specified by TSG alterations provide a framework for precision oncology in MPM.

## 1. Introduction

Malignant pleural mesothelioma (MPM) is a deadly cancer with incidence and mortality still increasing globally [1]. The leading cause for the poor prognosis of MPM is the extreme dearth of effective treatment options. The great majority of MPM patients present with advanced diseases, for whom a chemotherapy regimen (cisplatin plus pemetrexed) established in 2003 remains the only clinically approved first-line therapy [2].

Comprehensive genomic studies in MPM have revealed a rarity of pharmacologically tractable mutations in oncogenes [3,4,5], but the prevalence of inactivating alterations in tumor suppressor genes (TSGs), e.g., cyclin-dependent kinase inhibitor 2A/2B (*CDKN2A/2B*), BRCA1-associated protein-1 (*BAP1*), neurofibromin 2 (*NF2*), tumor protein p53 (*TP53*), SET domain containing 2 histone lysine methyltransferase (*SETD2*) and large tumor suppressor kinase 2 (*LATS2*). While the pharmacological inhibition of oncoproteins is successful, targeted therapies that exploit abnormal TSGs have proven far more difficult. Precision oncology, a burgeoning effort aimed at targeting unique molecular alterations of individual patients, has achieved great success in many cancers, but significantly lags behind in MPM. Consequently, clinical trials in MPM without biomarker-directed stratifications have generally failed [6,7,8,9].

Although the direct intervention of tumor suppressors is challenging, aberrant TSGs induce the reprogramming of biochemical networks, which creates cancer-specific vulnerabilities and provides an alternative venue for precision oncology in TSG-driven cancer [10]. Systematic correlation analysis is a powerful tool to identify rewired cellular processes, potential therapeutic targets, and associated biomarkers [11]. Here, by implementing weighted gene co-expression network analysis (WGCNA) [12], paralleled by comprehensive data mining and functional interrogation, we systematically delineated the biochemical networks induced by the inactivation of major TSGs (*CDKN2A/2B*, *BAP1*, *NF2*, *TP53*, *SETD2*, and *LATS2*) in MPM, and the underlying implications for precision oncology. Identification of molecular traits and the associated drug vulnerabilities co-selected by the functional loss of specific TSGs provides unprecedented insights into MPM pathobiology and may promote personalized treatment of MPM patients with molecularly guided, targeted- and immuno-therapy.

## 2. Results

### 2.1. Systematic Analysis of Rewired Biochemical Networks and Therapeutic Vulnerabilities Enabled by Tumor Suppressor Loss in MPM

**Figure 1:**
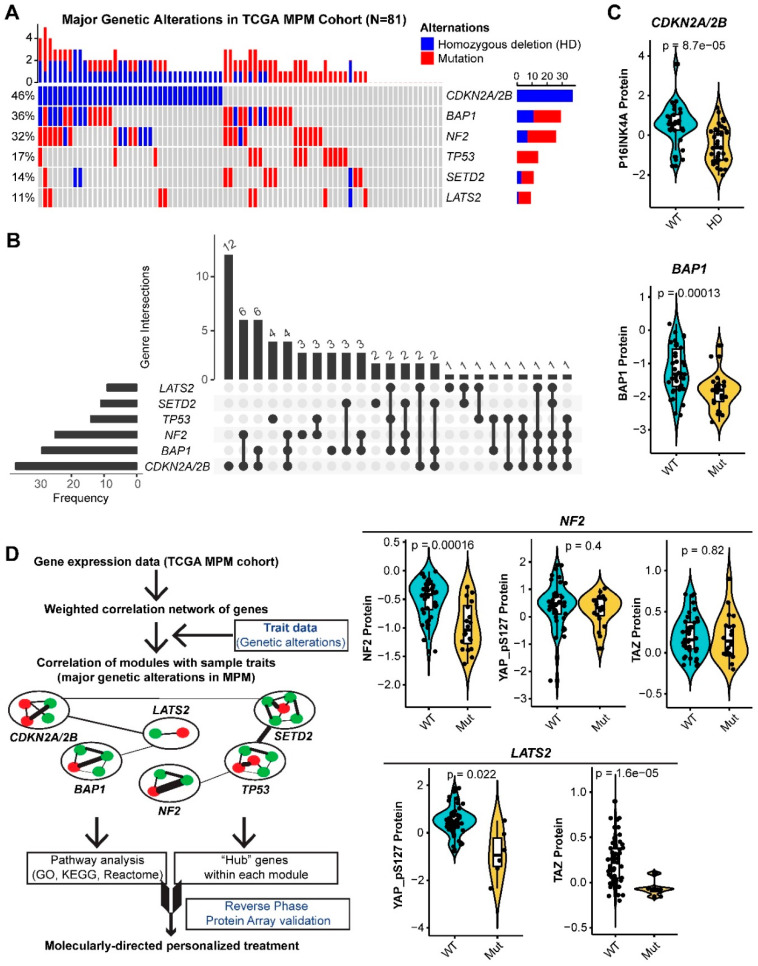
Major genetic alterations in The Cancer Genome Atlas (TCGA) MPM cohort. (**A**,**B**), Percentage (**A**) and overlap (**B**) of major (>10%) genetic alterations in The Cancer Genome Atlas (TCGA) malignant pleural mesothelioma (MPM) cohort (*N* = 81). (**C**), the association between the major genetic alterations (**A**) and the corresponding protein level in TCGA MPM cohort (*N* = 61). Protein array data were downloaded and reanalyzed from The Cancer Proteome Atlas (TCPA) database (https://tcpaportal.org/tcpa/). Of note, protein quantification data of LATS2 and SETD2 were not available in the TCPA database. Phospho-YAP (S127) and TAZ are two critical factors, indicating the activity of Hippo pathway. (**D**), Workflow of weighted gene correlation networks analysis (WGCNA).

All the major genetic alterations (>10%) occurring in TCGA MPM cohort are TSGs, including *CDKN2A/2B* (homozygous deletions (HDs)), *BAP1* (HDs and point mutations), *NF2* (HDs and point mutations), *TP53* (point mutations), *SETD2* (HDs and point mutations), and *LATS2* (HDs and point mutations) (Figure 1A). Notably, there are substantial overlaps of alterations in different TSGs (Figure 1B). For instance, the majority (67.6%) of the MPM tumors that harbor HDs of *CDKN2A/2B* have co-occurring alterations in other TSGs, e.g., *BAP1* (40.5%) or *NF2* (37.8%). Importantly, analyses of RPPA data of TCGA MPM cohort (*n* = 61) showed that genetic alterations remarkably decreased the levels of the encoded proteins or downstream effectors (Figure 1C).

To uncover fundamental molecular features associated with the functional loss of TSGs in MPM, we performed WGCNA, based on the transcriptomic data of TCGA MPM cohort (Figure 1D and Figure S1A–D), and delineated a network of multiple modules or clusters, that are significantly positively or negatively correlated with genetic inactivation of the top six TSGs in MPM (Figure S1E). Genes in the positively correlated modules indicate the abundance of the module-specified traits conferred by individual TSG loss, while those in the negatively correlated ones indicate the attenuation. Genes in the gray module are those that cannot be clustered.

### 2.2. CDKN2A/2B

*CDKN2A/2B* encodes three tumor suppressors, p16^INK4a^ and p14^ARF^ (by *CDKN2A*) and p15^INK4b^ (by *CDKN2B*), that play critical roles in cell cycle regulation. Moreover, p16^INK4a^ and p15^INK4b^ are functionally redundant by inhibiting cyclin-dependent kinase (CDK) 4/6 and cyclin D, and consequently blocking cell cycle progression from G1 to S [13].

**Figure 2:**
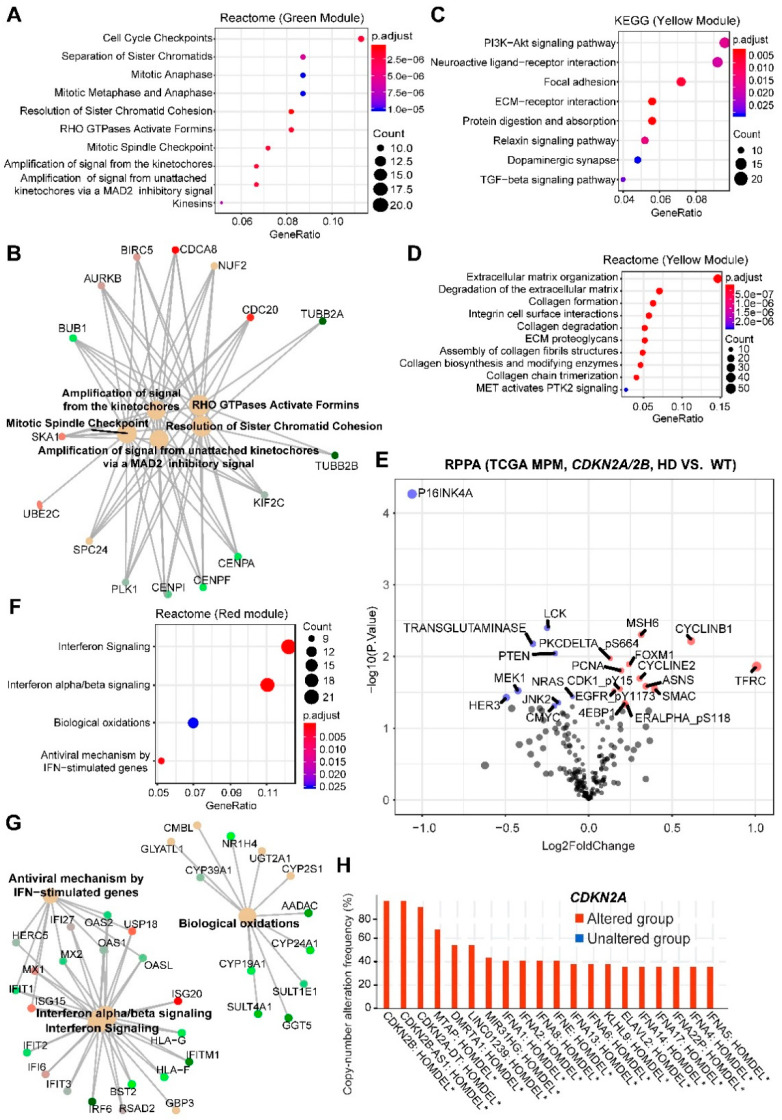
Enrichment analyses of genes significantly correlated with MPM tumors harboring HDs in *CDKN2A/2B*. (**A**,**B**), Top 10 significantly enriched Reactome pathways based on genes in the green module. In B, genes in the enriched Reactome pathways were listed. (**C**,**D**), Top 10 significantly enriched Kyoto Encyclopedia of Genes and Genomes (KEGG) (**C**) and Reactome (**D**) pathways based on genes in the yellow module. (**E**), Volcano plot showing the significantly (adjusted *p*-value < 0.05) upregulated (red) and downregulated (blue) proteins in malignant pleural mesothelioma (MPM) tumors harboring homozygous deletions (HDs) in CDKN2A/2B (versus wild-type), based on The Cancer Genome Atlas (TCGA) MPM cohort (*N* = 61). Data were downloaded and reanalyzed from The Cancer Proteome Atlas (TCPA) database (https://tcpaportal.org/tcpa/). (**F**,**G**), significantly enriched Reactome pathways based on genes in the red module. In (**G**), genes in the enriched Reactome pathways (**F**) were listed. (**H**), Genes significantly co-deleted with CDKN2A/2B in TCGA MPM samples. Data were downloaded from cBioPortal (https://www.cbioportal.org/). * *p* < 0.05. 2.3. BAP1.

The correlation network showed that *CDKN2A/2B* loss in MPM was significantly positively correlated with the green module (508 genes; correlation coefficient Pearson’s r = 0.55; *p*-value = 2 × 10^−7^, followed by the yellow (543 genes; *r* = 0.34; *p*-value = 0.002), but negatively with the red (356 genes; *r* = −0.36; *p*-value = 0.001) (Figure S1E). Pathway analyses (GO, KEGG, Reactome) revealed that the green module enriched the genes involved in cell cycle regulation, particularly checkpoints and mitosis (Figure 2A,B and Figure S2A), consistent with the function of *CDKN2A/2B* in cell-cycle regulation. The yellow module significantly enriched the genes of extracellular matrix (ECM)-receptor interaction, PI3K/AKT, and focal adhesion pathways (Figure 2C,D and Figure S2B,C). Interrogation of the RPPA data revealed that MPM deficient in *CDKN2A/2B* had significantly higher levels of proteins involved in the cell cycle (e.g., Cyclin B1, Cyclin E2, CDK1 (p-Y15), FOXM1) and PI3K (e.g., 4EBP1 and PKC-delta (p-S664)) pathways, but decreased p16^INK4a^ and PTEN (a negative regulator of PI3K) (Figure 2E), further supporting our results.

The red module negatively correlated with *CDKN2A/2B* loss enriched genes of anti-viral type I interferon (IFN-I, mainly IFN-α and IFN-β) signaling pathway, suggesting a link between *CDKN2A/2B* inactivation and impaired IFN-I pathway (Figure 2E–G and Figure S2D). To explore the underlying mechanisms, we analyzed co-occurring alterations in MPM samples, which revealed that *CDKN2A* and genes of the IFN family were significantly co-deleted (Figure 2H), consistent with a recent study, showing that defects in the IFN-I pathway mainly co-occur with *CDKN2A* loss [14].

We then analyzed intramodular connectivity, given that highly connected genes may serve as the hub with core regulatory roles. The top 20 best-connected genes in the green module are *KIF23*, *KIF4A*, *KIF2C*, *HJURP*, *KIF18B*, *MYBL2*, *BUB1*, *NUF2*, *UBE2C*, *CDCA8*, *CKAP2L*, *PLK1*, *DLGAP5*, *CDC20*, *TOP2A*, *DEPDC1*, *ANLN*, *CENPA*, *CDCA2*, *CEP55*. Most of these genes regulate the mitotic process and predict dismal prognosis in MPM (Figure S2E). Notably, the transcription factor MYBL2 is a central regulator of cell survival, proliferation and differentiation in cancer [15], and PLK1 and TOP2A are druggable by clinically advanced inhibitors. The top 20 best-connected genes in the yellow module are *COL5A1*, *VCAN*, *COL1A2*, *DACT1*, *FN1*, *CTHRC1*, *ITGA11*, *COL5A2*, *FAP*, *PODNL1*, *TGFB1I1*, *COL1A1*, *MMP2*, *COL3A1*, *LTBP1*, *MATN3*, *CHST6*, *POSTN*, *COL16A1*, *SRPX2*. Most of the genes are involved in ECM and associated with the suppression of anticancer immunity [16,17]. Supporting this notion, examining RPPA data revealed significantly decreased LCK, a key molecule in the selection and maturation of developing T-cells [18] (Figure 2E). Moreover, MPM has a high ECM signature compared to other solid tumors (Figure S3A), which predicts poor prognosis in patients (Figure S3B). However, the genetic underpinning for the high ECM of MPM has been unclear. Our data showed that the high ECM might be due to the high percentage (~46%) of MPM tumors with *CDKN2A/2B* alterations. The top 20 most connective genes in the red module are *OAS2*, *MX1*, *RSAD2*, *HERC6*, *IFIT3*, *CMPK2*, *IFI6*, *ISG15*, *USP18*, *IFIT2*, *OASL*, *IFI44*, *MX2*, *DDX60*, *IFI44L*, *OAS1*, *LAMP3*, *CYP39A1*, *IFIT1*, *RUFY4*, with the vast majority involved in the IFN-I pathway.

Collectively, these results reveal cellular processes that may represent therapeutic vulnerabilities in *CDKN2A/2B* deficient MPM. The enriched green and yellow modules indicate that *CDKN2A/2B*-mutant MPM may benefit from the co-targeting of the G2/M checkpoint or mitosis (e.g., PLK1) with PI3K/AKT, but might be associated with suppressive anticancer immunity due to high ECM. Oncolytic viral immunotherapy, a novel anticancer strategy preferentially killing proliferating cancer cells but sparing normal ones, might be particularly effective for the red module-marked subset, in which the IFN-I pathway genes are often co-deleted.

BAP1 has pleiotropic roles, ranging from the maintenance of genomic stability to the repair of DNA double-strand breaks (DSBs) [19,20]. Our analysis showed that *BAP1* alterations in MPM are positively correlated with the red module only (*r* = 0.41; *p*-value = 2 × 10^−4^) that enriches the IFN-I pathway (Figure 2F,G), and negatively correlated with *CDKN2A/2B* loss (Figure S1E). This finding is supported by our recent study, showing that *BAP1* is negatively correlated with the IFN-I gene signature [21]. Thus, *CDKN2A/2B* deficiency plus *BAP1* proficiency defines a unique MPM subset that might particularly be sensitive to oncolytic viral immunotherapy.

### 2.3. NF2

NF2 is a plasma membrane protein binding to α-catenin and tight junctions to suppress cell growth. NF2 loss deregulates multiple signal pathways, although a prevalent notion holds that the Hippo pathway is central to the phenotype of *NF2*-mutant MPM.

Akin to *CDKN2A* loss, *NF2* alterations are positively correlated with the green (*r* = 0.34; *p*-value = 0.002) and the yellow (*r* = 0.26; *p*-value = 0.02) modules (Figure S1E), suggesting that NF2 might regulate cell cycle [22,23] and PI3K/AKT/mTORC1 (yellow module) [24], in addition to the canonical Hippo pathway. Supporting the notion, mining the public dataset that elaborates on protein-protein interactions revealed that the proteins involved in the ribosome, tight junction, Hippo and DNA repair are enriched in NF2-binding partners (Figure S4). The similarity between *CDKN2A-* and *NF2-*associated gene expression can alternatively be because *CDKN2A* and *NF2* alterations overlap in MPM (Figure 1B). However, *CDKN2A* and *BAP1* deficiency co-occurs at an even greater extent (Figure 1B) but rewires different gene networks (Figure 2) argues against this possibility.

Thus, like *CDKN2A/2B*, the genetic inactivation of *NF2* deregulates cell cycle, ECM and PI3K/AKT pathways, which prioritizes the co-targeting of the G2/M checkpoint/mitosis and PI3K/AKT pathway for *NF2*-altered MPM.

### 2.4. TP53

*TP53* mutations are negatively correlated with the purple module (125 genes; *r* = −0.37; *p*-value = 9 × 10^−4^), to a less extent with the turquoise (1143 genes; r = −0.29; *p*-value = 0.01) and the green-yellow (108 genes; *r* = −0.27; *p*-value = 0.02), but positively with the salmon (57 genes; *r* = 0.23; *p*-value = 0.04), implying that *TP53* mutations deregulate multiple biological processes in MPM (Figure S1E). Notably, the turquoise is also significantly correlated with *LATS2* alterations (Figure S1E); we therefore focused on the purple and green-yellow module in the context of *TP53* mutations.

**Figure 3:**
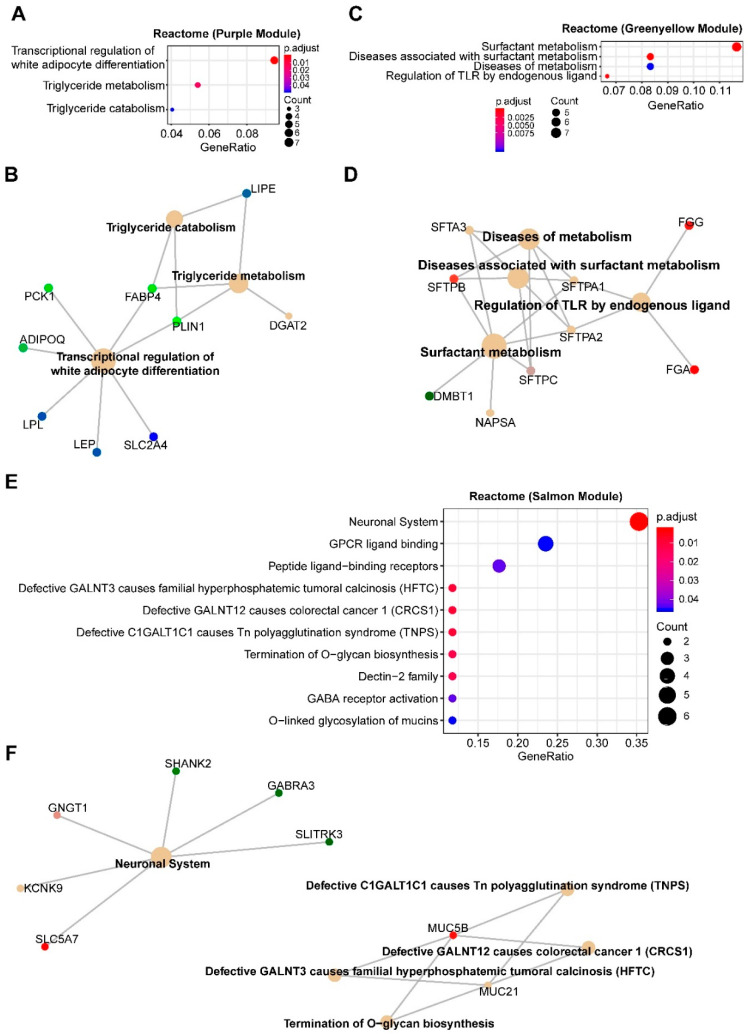
Enrichment analyses of genes significantly correlated with MPM tumors with TP53 alterations. (**A**,**E**), Significantly enriched Reactome pathways based on genes in the purple (**A**,**B**), green-yellow (**C**,**D**) and salmon (**E**,**F**) modules. Cnetplots in (**B**), (**D**) and (**F**) listed genes in the enriched Reactome pathways (**A**, **C** and **E**, respectively).

The purple module enriches genes of adipocyte differentiation/lipid metabolism, suggesting that *TP53*-mutant MPM might have attenuated activity of the processes (Figure 3A,B and Figure S5A) and benefit from differentiation therapy, e.g., peroxisome proliferator-activated receptor (PPAR) activator (Figure S5A). Supporting this notion, PPAR activator has been shown to promote the differentiation of mesenchymal therapy-resistant cancer cells to adipocytes [25]. Furthermore, the green-yellow module negatively correlated with *TP53* mutations enrich genes involved in lung epithelial cell differentiation (Figure 3C,D and Figure S5B), and the positively correlated salmon module enriches for genes of the neuronal system (Figure 3E,F). However, the marginal significance (*p*-value = 0.04) limits the value of this module.

The top 20 best-connected genes within the purple module are AQP7, PLIN1, ADIPOQ, TUSC5, CIDEA, THRSP, PLIN4, CIDEC, C14orf180, AQP7P1, CD300LG, C6, LIPE, LEP, NTRK2, SLC7A10, KCNIP2, GPD1, PDK4, and LPL, among which chemical agonists for PDK4, PRKAR2B and LPL are available. The top 20 best-connected genes of the green-yellow module include PDK4, TUSC5, LIPE, CIDEC, KCNIP2, CTSG, THRSP, CIDEA, AQP7P1, CD300LG, C7, C6, FREM1, THSD7B, MS4A2, TPSB2, C14orf180, FAM107A, TPSAB1, and TNMD.

### 2.5. SETD2

*SETD2* is a histone-modifying enzyme responsible for trimethylation of the lysine 36 residue on Histone 3 (H3K36me3) in humans. Impaired H3K36me3 causes aberrant gene regulation and chromosomal instability [26].

**Figure 4:**
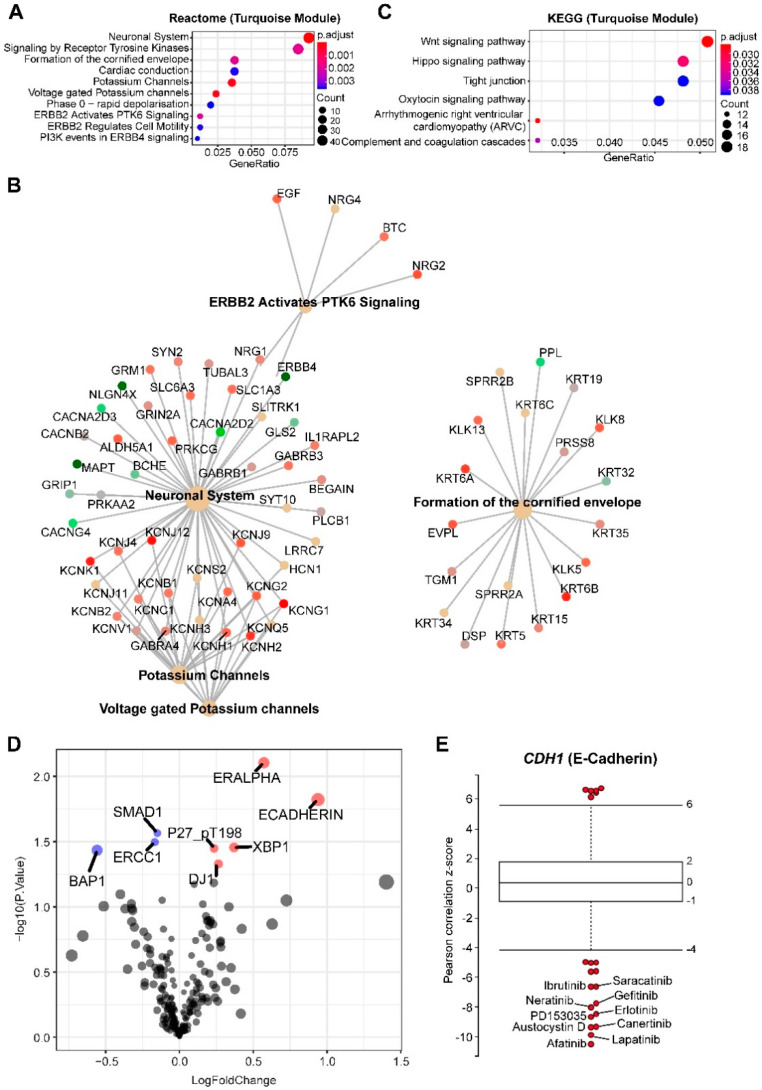
Enrichment analyses of genes significantly correlated with MPM tumors with *SETD2* alterations. (**A**,**C**) Top 10 significantly enriched Reactome (**A**,**B**) and Kyoto Encyclopedia of Genes and Genomes (KEGG) (**C**) pathways based on genes in the turquoise module. Cnetplot in (**B**) listed genes in the enriched Reactome pathways (**A**). (**D**) Volcano plot showing the significantly (adjusted *p*-value < 0.05) upregulated (red) and downregulated (blue) proteins in malignant pleural mesothelioma (MPM) tumors with SETD2 alterations (versus wild-type), based on The Cancer Genome Atlas (TCGA) MPM cohort (*N* = 61). Data were downloaded and reanalyzed from The Cancer Proteome Atlas (TCPA) database (https://tcpaportal.org/tcpa/). (**E**) Box-and-whisker plots show the extent of correlation between cytotoxic effects of each compound and with CDH1 (encoding E-cadherin) mRNA level, across 670 solid cancer cell lines. The y-axis indicates z scored Pearson’s correlation coefficients; line, median; box, 25–75th percentile; whiskers, 2.5th and 97.5th percentile expansion; Here, only significantly (*p* < 0.05) correlated inhibitors were shown (in red dots). Labeled dots indicated the most negatively correlated drugs.

MPM with *SETD2* alterations is exclusively abundant (*r* = 0.25; *p*-value = 0.03) in the turquoise module, consisting of 1143 genes, with functions spanning from neuronal biology and receptor tyrosine kinases (particularly EGFR family) to the potassium channel, the Hippo and Wnt (Figure 4A–C). The Hippo and Wnt pathways are tumor-suppressive, precluding the potential as therapeutic targets. However, our results suggest that targeting EGFR might be a novel strategy for *SETD2*-altered MPM (Figure 4A,B).

Genetic/molecular co-occurrence in tumor samples implies that progression to malignancy is a consequence of cooperative genetic/molecular dysregulations. Indeed, genetic alterations in *EGFR* and *SETD2* frequently co-occur in glioma [27] and TCGA pan-cancer cohort (Figure S6), supporting the notion that co-occurring *EGFR* and *SETD2* alterations cooperate to promote tumor progression, and that *SETD2*-mutant cancer may evolve a dependency on EGFR signaling. To further confirm the link between *SETD2* alterations and sensitivity to EGFR inhibition, we performed integrated analyses of proteomic (RPPA) and drug sensitivity data, which revealed that E-cadherin is significantly upregulated in *SETD2*-altered MPM (Figure 4D) and the expression of *CDH1* (encoding E-cadherin) is most negatively correlated with sensitivity to various EGFR inhibitors (Figure 4E). Of note, the red module, abundant in the IFN-I signature and positively correlated with *BAP1* alterations, is also positively correlated with *SETD2* mutations in MPM. This can be explained by considerably co-occurring *BAP1* and *SETD2* mutations, as 8 of 11 *SETD2*-altered MPM also have aberrant *BAP1* (Figure 1B). RPPA analysis confirmed significantly downregulated BAP1 in *SETD2*-altered MPM (Figure 4D).

The top 20 best-connected genes in the turquoise module are KLK11, CCDC64, CARNS1, CGN, BNC1, CLDN15, COBL, PARD6B, PLLP, PRR15, IGSF9, PRR15L, ANXA9, SELENBP1, PDZK1IP1, TGM1, SOX6, HOOK1, MSLN, NRG4. One of the hub genes in this module is MSLN, encoding mesothelin, a well-characterized biomarker for mesothelial tissue, and commonly overexpressed in epithelial mesotheliomas.

### 2.6. LATS2

At the heart of the Hippo pathway stands a core kinase cassette: MST1/2, LATS1/2, and adaptor proteins SAV1, MOB1A/B, which converges at LATS1/2-dependent phosphorylation of Yes-associated protein (YAP) and transcriptional co-activator with TAZ.

**Figure 5:**
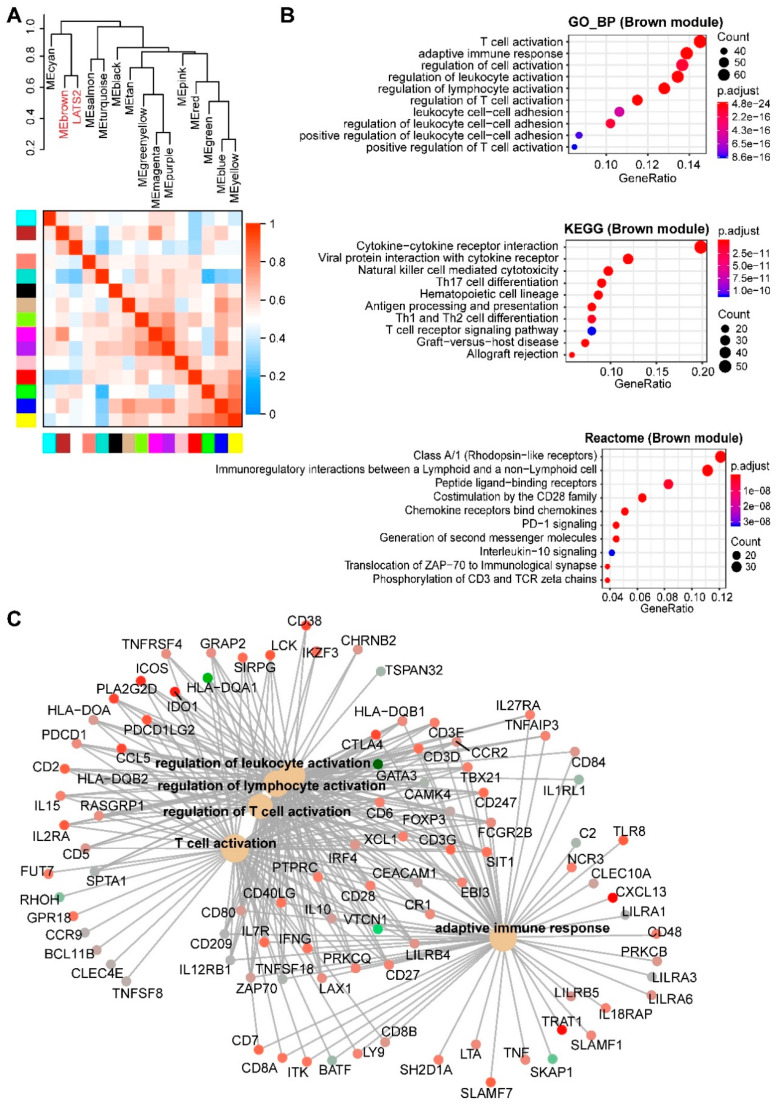
Enrichment analyses of genes significantly correlated with MPM tumors with LATS2 alterations. (**A**), Hierarchical clustering dendrogram of module eigengenes (labeled by their colors) and the sample trait (genetic alterations). Heatmap plot of the adjacencies in the eigengene network. In the heatmap, each row and column corresponds to one module eigengene (labeled by colors) or the trait. In the heatmap, green color indicates a negative correlation, while red represents a positive correlation. (**B**,**C**), Top 10 significantly enriched GO (biological process, BP), Kyoto Encyclopedia of Genes and Genomes (KEGG) and Reactome (**C**) pathways based on genes in the brown module. Cnetplot in C listed genes in the enriched Reactome pathways (**B**).

**Figure 6:**
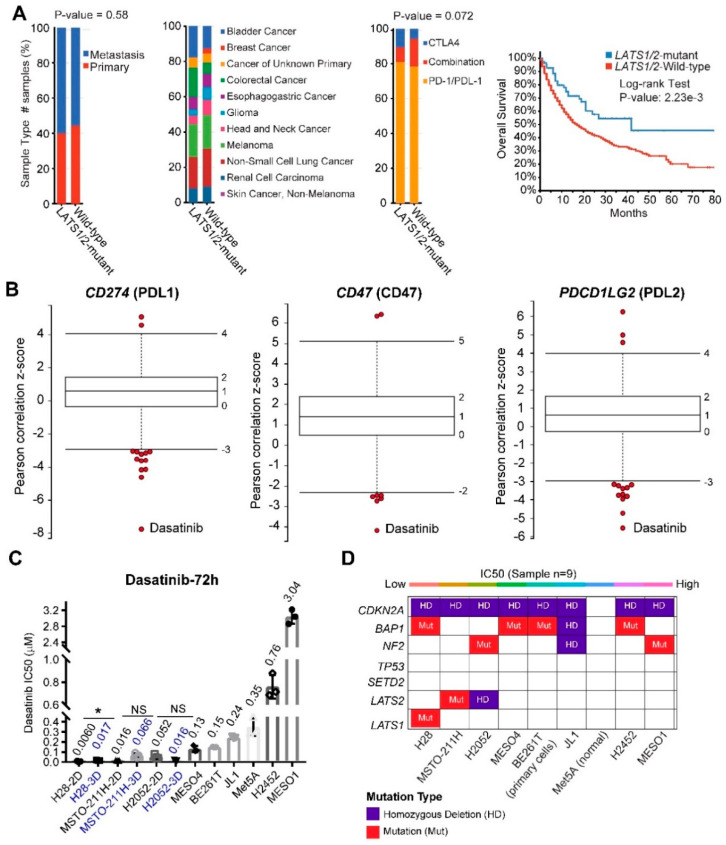
Identify Dasatinib as a promising therapeutic drug for MPM with *LATS2* alterations. (**A**) *LATS1/2* mutational status is associated with significantly improved overall survival in cancer patients after immune checkpoint blockage. The distribution of sample type (primary vs. metastatic; left panel), cancer type (middle panel) and drug type (anti-CTLA4; anti-PD1/PDL1; right panel) between *LATS1/2*-mutant and wild-type cancer. (**B**) Box-and-whisker plots show the extent of correlation between cytotoxic effects of Dasatinib and with several well-characterized immune markers (PDL1, PDL2, CD47), preferentially expressed by cancer cells. The y-axis indicates z scored Pearson’s correlation coefficients; line, median; box, 25–75th percentile; whiskers, 2.5th and 97.5th percentile expansion; Here, only significantly (*p* < 0.05) correlated inhibitors were shown (in red dots). Notably, Dasatinib is the most negatively correlated drug. (**C**,**D**) the median inhibitory concentration (IC50) values of a panel of MPM cell lines treated with Dasatinib (72 h). MPM cells seeded in triplicate at 96-well plates were drugged 24 h later, over a 12-point concentration range (two-fold dilution). DMSO-treated cells were used as control. IC50 was determined using GraphPad Prism 7. IC50 values of Dasatinib in three MPM cell lines (H28, MSTO-211H, H2052) cultured in 2D and 3D were compared. * *p* < 0.05 by Welch’s *t*-test. *N* = 3 biological replicates. In D, the genetic annotations of MPM cell lines (**C**) were shown.

*LATS2* alterations show a negative correlation with the turquoise module (Figure 2, *r* = −0.45; *p*-value = 4 × 10^−5^), which is opposite to *SETD2* alterations (positively correlated with the turquoise), but expected, in that genes involved in the Hippo and tight junction pathways are enriched in the turquoise module. Importantly, *LATS2* alterations in MPM are exclusively positively correlated (*r* = 0.33; *p*-value = 0.004) with the brown module (Figure S1E and Figure 5A), which significantly enriches for genes involved in immunoregulation (Figure 5B,C). These results suggest an immunoregulatory role beyond the canonical Hippo pathway by LATS2 and a rationale of immunotherapy for *LATS2*-altered MPM. Supporting the notion, PD-L1 (encoded by *CD274*) is the most significantly upregulated protein in *LATS2*-mutant MPM (Figure S7A), and LATS1/2 deletion has recently been shown to enhance anti-tumor immune responses [28]. Strikingly, a retrospective analysis of patients after being treated with immune checkpoint blockade showed that mutations of *LATS1/2*, rather than of *NF2*, predict significantly better survival (Figure 6A and Figure S7B).

The top 20 best-connected genes in the brown module are LCK, CD3E, IL2RG, SLAMF6, CD2, CD3D, SIT1, SH2D1A, CXCR3, TIGIT, TRAT1, CD6, GZMK, CD247, SIRPG, CD27, ZAP70, TBC1D10C, CD96, CD5. Of these, CD3E, IL2RG, CD2, CD3D, CD6, CD247, CD5, ITK, and CD3G are pharmacologically tractable.

Protein domains are important functional units and crucial for deconvolution of drug targets; we thus explored functional domains of the proteins encoded by the top 20 hub genes. Using SMART and PFAM protein fomains, we found that immunoreceptor tyrosine-based activation motif and Src homology 2 (SH2) domains are significantly enriched (false discovery rate < 0.05) in the hub proteins (Figure S7C). By correlating drug sensitivity with the gene expression of cancer cell lines (*n* = 670), we identified Dasatinib, a potent Abl/Src inhibitor, with the efficacy negatively correlated with several immune biomarkers (*CD274*, *CD47*, *PDCD1LG2*), that are preferentially expressed by cancer cells (Figure 6B). These results suggest that a role by LATS2 in cancer immunity and the potential of Dasatinib to target *LATS2*-altered MPM.

As preclinical proof of the concept, we found that *LATS1/2*-altered MPM cells exhibited the highest sensitivity to Dasatinib (Figure 6C,D). Importantly, the *LATS1/2*-altered MPM cells cultured in 3D retain a high sensitivity to Dasatinib (Figure 6C). Surprisingly, the mutational status of NF2, an upstream factor of LATS1/2 in the Hippo pathway, appeared not to predict the sensitivity to Dasatinib, which may suggest that NF2 and LATS1/2 have distinct and uncoupled functions in MPM. Further supporting our finding, Dasatinib was reported to show durable anticancer effects by promoting anti-tumor T cell responses, besides direct targeting of Abl/Src [29,30].

Finally, by analyzing RPPA data, we identified several antioxidant and anti-ferroptotic proteins, e.g., TFRC, GP6D, and PRDX1, that are significantly enriched in *LATS2*-altered MPM (Figure S7) [31]. In line with this observation, MPM with the aberrant Hippo pathway was reported to be susceptible to ferroptosis induction [32].

These results uncover an unexpected role for LATS2 in modulating immune contexture, suggesting a rationale for Dasatinib to treat *LATS2*-mutant MPM. Our data also argue that LATS2 and NF2 may exert distinct roles in MPM, at odds with the long-held assumption that they act as tumor suppressors through the Hippo pathway.

## 3. Discussion

Cancer patients vary in prognosis and response to therapy due to tumor heterogeneity [33,34], highlighting the need for personalized treatment. Unlike many other solid tumors, MPM is characterized by a pharmacologically intractable abnormal tumor genome, mainly TSGs, for which targeted therapy has been poorly established. In this study, we presented, for the first time, a systematic analysis of biochemical networks and associated vulnerabilities induced by the functional loss of TSGs in MPM, which not only sheds light on the mechanisms of MPM biology but also provides a framework of biomarker-guided targeted therapy in MPM (Figure S8).

### 3.1. CDKN2A/2B and NF2

An important finding of this study is that *CDKN2A* and *NF2* loss leads to similar changes in cellular pathways in MPM. Despite the evidence for targeting PI3K/AKT/mTOR pathway in MPM subsets [3,35,36,37,38], whether the deregulation of the pathway is associated with specific genetic events is unclear. Our results reveal the molecular underpinning of *CDKN2A* and *NF2* deficiencies, and further suggest therapeutic options for these MPM subsets. As p16INK4a (product of *CDKN2A*) inhibits CDK4/6 [13], CDK4/6 activation upon *CDKN2A* loss renders *CDKN2A*-deficient MPM particularly vulnerable to CDK4/6 inhibitors [36,39], and co-targeting CDK4/6 and PI3K/AKT/mTOR induce synergistic anti-MPM effects [36]. PI3K/mTOR inhibitors as monotherapy failed in unselected MPM patients [7], highlighting the importance of biomarker-guided stratification in future clinical trials.

Oncolytic viral immunotherapy shows promises in MPM [40], partly due to the special location of the malignancy that facilitates viral administration. We showed that IFN-I pathway genes are often co-deleted with *CDKN2A*, suggesting a rational by oncolytic viral immunotherapy for *CDKN2A*-altered MPM, which is supported by a recent report [14]. As *CDKN2A/2B* loss is widely used in pathological diagnosis to distinguish MPM from benign pleural lesions, analyzing the mutations of IFN-I–related genes will improve MPM diagnosis and patient stratification.

MPM has a high ECM signature, which may drive immunotherapy resistance [16,17]. Here, we provided evidence that high ECM in MPM is mainly attributable to *CDKN2A/2B* and *NF2* deficiency, that accounts for ~55.6% (45 of 81) of MPM cases (Figure 1B).

### 3.2. BAP1

BAP1 loss is frequent in MPM, renal cell carcinoma, peritoneal mesothelioma, and uveal melanoma [41]. Given the role of BAP1 in the maintenance of genomic stability, the association between *BAP1* mutations and sensitivity to PARP1-targeted therapy has been demonstrated in the chicken model of DT40 cells [19]. However, we and others have recently shown that *BAP1* mutations cannot precisely predict the response to PARP1-targeted therapy in MPM [20,42]. In addition, BAP1 status has been shown to determine the sensitivity to Gemcitabine treatment in MPM [43,44]. Here, *BAP1* alterations show significant abundance in IFN-I pathway only, consistent with our finding that *BAP1* is negatively correlated with the IFN-I signature in MPM [21]. Our data suggest that *CDKN2A* deficiency and *BAP1* proficiency should be considered to stratify MPM for oncolytic viral immunotherapy.

### 3.3. TP53

Mutant p53 has been proposed to drive metabolic reprogramming, thereby promoting cancer progression [45,46,47,48]. Our data reveal a potential role for *TP53* mutation in lipid metabolism, by deregulating the PPAR signaling pathway. Supporting our finding, p53 interacts with PPAR-γ co-activator 1α (PGC-1α) [45,46,47], and PPAR activator promotes the differentiation of mesenchymal therapy-resistant breast cancer cells [25]. These results warrant further studies to test differentiation therapy for *TP53*-mutant MPM.

Notably, synthetic lethal targets with p53 inactivation have been investigated [49,50,51]. In particular, MDM2, a nuclear E3 ubiquitin ligase that binds and targets p53 for proteasomal degradation, is detected in 21.3% of clinical MPM samples, and its expression is significantly associated with poor survival [52]. To restore p53 function, several small molecules, such as the Nutlin-like drugs that disrupt MDM2/p53 interaction, have been tested in MPM [53,54,55]. Moreover, we and others have shown that the inactivation of *CDKN2A/2B* and *TP53* is associated with an increased dependence on the G2/M checkpoint, which represents a targetable vulnerability in MPM [56,57].

### 3.4. SETD2

We showed that SETD2 might have roles beyond histone modifications. Of note, RTKs, particularly EGFR members (HER1 (EGFR, ERBB1), HER2 (NEU, ERBB2), HER3 (ERBB3), and HER4 (ERBB4)) were exclusively enriched in *SETD2*-altered MPM, suggesting the potential of pan-EGFR inhibitors for this MPM subset. Indeed, co-mutant *EGFR* and *SETD2* are common in glioma and pan-cancer [27], suggesting that *SETD2*-mutant cancer might have evolved a unique dependence on EGFR signaling.

EGFR is not mutated, but overexpressed in MPM [58,59,60]. A previous study showed that MPM expressed EGFR (79.2%), ErbB4 (49.0%) and HER2 (6.3%), but lacked ErbB3 [61]. In line with this, anti-HER-2 antibody synergizes with cisplatin in a subset of MPM cell lines [62]. However, the first-generation EGFR/ERBB1 inhibitor erlotinib [9] and gefitinib [8] show no clinical benefit, suggesting that pan-EGFR inhibitors might be necessary. To be noted, EGFR and other RTKs (MET, AXL) have been demonstrated to contribute to the activation of the downstream PI3K/AKT/mTOR in MPM [35], and the targeting PI3K/AKT/mTOR pathway, alone or in combination with other agents, have been investigated in MPM [7,36,37,38]. We showed that E-Cadherin is overexpressed in *SETD2*-altered MPM and predicts the sensitivity to EGFR-targeted therapies. Our finding that E-cadherin is significantly negatively correlated with EGFR inhibitor efficacy prioritizes the need for biomarker-driven selection and pan-EGFR inhibitors that target ERBB2/3/4 as well.

### 3.5. LATS2

LATS1/2 are key players of the Hippo pathway, but only LATS2 is frequently mutated in MPM. We identified the significant enrichment of immunoregulatory pathways in *LATS2*-mutant MPM, suggesting an unanticipated role for LATS2 in immunoregulation. Supporting our finding, LATS1/2 can suppress cancer immunity, and their deletion improves tumor immunogenicity by enhancing anti-tumor immune responses [28]. These results support a rationale of immunotherapy to target *LATS2*-altered MPM, although how LATS1/2 modulates the immune response awaits further studies.

Immunotherapy shows promises in MPM, but with low and heterogeneous response rates [63,64], arguing for biomarker-guided stratifications of MPM subsets responsive to immunotherapies. Our data suggest that *LATS2* mutational status might be a critical factor in selecting MPM patients who can benefit from immunotherapies.

Strikingly, our study identified Dasatinib, a clinically approved RTK inhibitor, as a promising therapeutic for *LATS2*-altered MPM. Dasatinib shows the potential to modulate anticancer immunity (Figure 6B), and selectively impairs *LATS2*-altered MPM cells (Figure 6C), in line with the evidence that Dasatinib enhances anti-PDL1 efficacy in cancer [30]. These data suggest a rationale, by combining Dasatinib with immune checkpoint blockades to treat *LATS2*-altered MPM. Indeed, *LATS2* mutations are associated with beneficial survival in immunotherapy-treated patients (Figure 6A), but Dasatinib as monotherapy failed in unselected MPM patients [6,65], supporting the use of *LATS2* mutational status for patient stratification in clinical trials with Dasatinib.

Finally, we reveal a significant enrichment of proteins regulating ferroptosis in *LATS2*-mutant MPM, but not in those with *NF2* alterations, which is at odds with a recent report, showing that aberrant NF2-Hippo pathway is selectively susceptible to ferroptosis induction [32]. The observation that NF2 and LATS2 likely play different roles in MPM is supported by several lines of evidence. First, *LATS2* rather than *NF2* alterations are associated dysregulated YAP and TAZ (Figure 1C); secondly, *LATS2*- and *NF2-*mutant tumors show strikingly different enrichment of gene and protein signatures (Figures S1E and S7 and Figure 5); thirdly, Dasatinib selectively impairs *LATS2-* but not *NF2*-altered MPM (Figure 6); fourthly, *LATS1/2* mutations but not *NF2* alterations predict better survival in patients after immune checkpoint blockade therapy (Figure 6A and Figure S7B). Together, our data suggest that LATS2 and NF2 might have distinct roles in MPM, despite the long-held notion that both function through the Hippo pathway.

## 4. Materials and Methods

### 4.1. WGCNA and Function Enrichment Analyses

To identify the gene expression profiling associated with the major genetic alterations in MPM, The R package “WGCNA” was applied to the RNA-sequencing data retrieved from TCGA MPM cohort. In WGCNA, genes are clustered based on co-expression patterns to construct a gene co-expression network, which was transformed into the adjacency matrix and then topological overlap matrix (TOM) [12]. According to the TOM-based dissimilarity measure, genes were grouped into different modules (clusters) using the dynamic tree cut algorithm. For each module, the module eigengene (ME) was calculated; the first principal component representative of the module. The ME values were correlated with sample traits defined by specific genetic alterations in MPM samples. Here, we set the soft-thresholding power at 5 (scale-free R2 = 0.86), cut height at 0.25, and minimal module size to 30, to identify key modules. The module significantly correlated with sample traits was selected to explore its biological functions, such as gene ontology (GO), Kyoto Encyclopedia of Genes and Genomes (KEGG) and reactome pathway enrichment analyses, using the R package “clusterprofiler” [66]. Hub genes were defined as top 20 intramodular connected genes.

### 4.2. Cell Viability Assay

All normal human mesothelial cells Met-5A (MeT-5A, RRID: CVCL_3749), MPM cell lines H28 (NCI-H28, RRID: CVCL_1555), H2452 (NCI-H2452, RRID: CVCL_1553), and H2052 (NCI-H2052, RRID: CVCL_1518) were obtained from ATCC (American Type Culture Collection, Manassas, VA, USA) [67]. MPM cell lines MESO-1 (ACC-MESO-1, RRID: CVCL_5113) and MESO-4 (ACC-MESO-4, RRID: CVCL_5114) were obtained from RIKEN Cell Bank (Ibaraki, Japan). MPM cell lines MSTO-211H (RRID: CVCL_1430) and JL-1 (RRID: CVCL_2080) were purchased from DSMZ (German Collection of Microorganisms and Cell Cultures, Brunswick, Germany). A primary MPM cell culture (BE261T) was established from surgically resected tumors of a 67-year-old male patient, using the same protocol as described in [67] and used for short-term studies (up to eight passages in vitro). The human study was performed under the auspices of protocols approved by institutional review board (KEK number: 042/15), and informed consent was obtained from patients. Cells were cultured in RPMI-1640 medium (Cat. #8758; Sigma-Aldrich, St. Louis, MO, USA), supplemented with 10% fetal bovine serum/FBS (Cat. #10270-106; Life Technologies, Grand Island, NY, USA) and 1% penicillin/streptomycin (P/S) solution (Cat. #P0781, Sigma-Aldrich, St. Louis, MO, USA). For 3D culture, cells were cultured in ultra-low attachment plate (Sigma-Aldrich, #CLS3474-24EA) with FBS-free RPMI-1640 medium supplemented with EGF (20 ng/mL; Cat. #PHG0311; Thermo Fisher Scientific (Waltham, MA, USA), bFGF (20 ng/mL; Cat. #PHG6015; Thermo Fisher Scientific), 4µg/mL insulin (Cat. #I9278; Sigma-Aldrich), 1× B-27 (Cat. #17504044; Thermo Fisher Scientific), 1% P/S. All human cell lines have been authenticated using STR profiling within the last three years, and are confirmed free from mycoplasma contamination (Microsynth, Bern, Switzerland).

MPM cells seeded in triplicate at 96-well plates (for 2D: 1000–1500 cells/well in tissue-culture treated plate (Corning, #353072); for 3D: 4000–5000 cells/well in ultra-low attachment plate) were drugged 24 h later, over a 12-point concentration range (two-fold dilution), with DMSO as vehicle. Cell viability was determined 72 h post-treatment by the Acid Phosphatase Assay Kit (ab83367; Abcam) [68]. The median inhibitory concentration (IC50) was calculated using GraphPad Prism 7.

### 4.3. Public Databases

RNA-sequencing data of MPM samples (*n* = 87) were downloaded from TCGA (https://portal.gdc.cancer.gov/), in which 81 samples were provided with genetic alterations data. Normalized level 4 data of reverse phase protein array (RPPA) were downloaded from The Cancer Proteome Atlas (TCPA) database (https://tcpaportal.org/tcpa/) [69], which quantified 218 proteins in 61 out of the 87 MPM samples in TCGA. R packages “limma” and “edgeR” were used to normalize the data and identify the differential gene or protein expression, respectively [70]. Protein-interacting data were downloaded from Agile Protein Interactomes DataServer (http://cicblade.dep.usal.es:8080/APID/init.action) [71], and co-occurring analysis data were downloaded from cBioPortal (https://www.cbioportal.org/). Processed drug (*n* = 481) screening and gene expression data across solid cancer cell lines (*n* = 659) were downloaded and reanalyzed from a published study [11]. Fisher’s z-transformation was applied to the correlation coefficients to adjust for (normalize) variations in cancer cell line numbers across small molecules and cell lineages. Genetic and survival data of patients after immunotherapies (anti-PD1/PDL1, anti-CTLA4) were from TMB and immunotherapy (MSKCC) cohort in cBioPortal [72].

### 4.4. Survival Analysis

Survival analysis was performed using “survminer” and “survival” R packages. Tumor samples within the TCGA MPM cohort were divided into two groups, based on each hub gene’s best-separation cut-off value to plot the Kaplan–Meier survival curves.

### 4.5. ECM Gene Signature

The extracellular matrix (ECM)/stromal gene signature was scored as the sum of an ECM/stromal gene set (*VCAN*, *FAP*, *POSTN*, *FBLN1*, *COL1A1*, *PDPN*, *THY1*, *CSPG4*, *IL6*, *TGFB1*, *HGF*, *SERPINE1*). The gene list was curated based on previous studies across different cancer lineages [16,17].

### 4.6. Statistical Analysis

Data were presented as mean ± SD, with the indicated sample size (*n*) representing biological replicates. Gene expression and survival data derived from the public database, as well as the correlation coefficient, were analyzed using *R* (version 3.6.0). *p* < 0.05 was considered statistically significant.

## 5. Conclusions

Overall, we report the systematic identification of biochemical networks and therapeutic potential linked with aberrant TSGs, which provides a framework for biomarker-guided precision oncology for MPM subsets. Our work warrants further studies that verify the drug vulnerabilities and the stratification approaches for future clinical trials.

## Supplementary Materials

The following are available online at https://www.mdpi.com/2072-6694/12/8/2310/s1, Figure S1: Weighted gene correlation network analysis (WGCNA) reveal gene modules linked with major genetic alterations in MPM; Figure S2: Pathway enrichment analyses of the genes significantly correlated with CDKN2A/2B loss; Figure S3: MPM has a high extracellular matrix (ECM) gene signature; Figure S4: Pathway enrichment analyses of the genes significantly correlated with NF2 alterations; Figure S5: Pathway enrichment analyses of the genes significantly correlated with TP53 mutations; Figure S6: Mutually exclusive and co-occurring analyses of STED2 and EGFR family genes across TCGA pan-cancer solid tumors; Figure S7: LATS2-altered MPM tumors enrich for the immune-regulatory signature; Figure S8: Tumor suppressor genes (TSGs)-guided precision oncology in MPM.
