# Supplementary material for: Systematic Analysis of Aberrant Biochemical Networks and Potential Drug Vulnerabilities Induced by Tumor Suppressor Loss in Malignant Pleural Mesothelioma
Source: Cancers (Basel). 2020 Aug 17;12(8):2310. doi: 10.3390/cancers12082310 (PMC7465812; doi:10.3390/cancers12082310)
Supplement: Supplementary file 1 [file cancers-12-02310-s001.pdf]

Supplementary Materials: Systematic Analysis of Aberrant Biochemical Networks and Potential Drug Vulnerabilities Induced by Tumor Suppressor Loss in Malignant Pleural Mesothelioma

Haitang Yang Duo Xu, Zhang Yang, Feng Yao, Heng Zhao, Ralph A. Schmid and Ren-Wang Peng

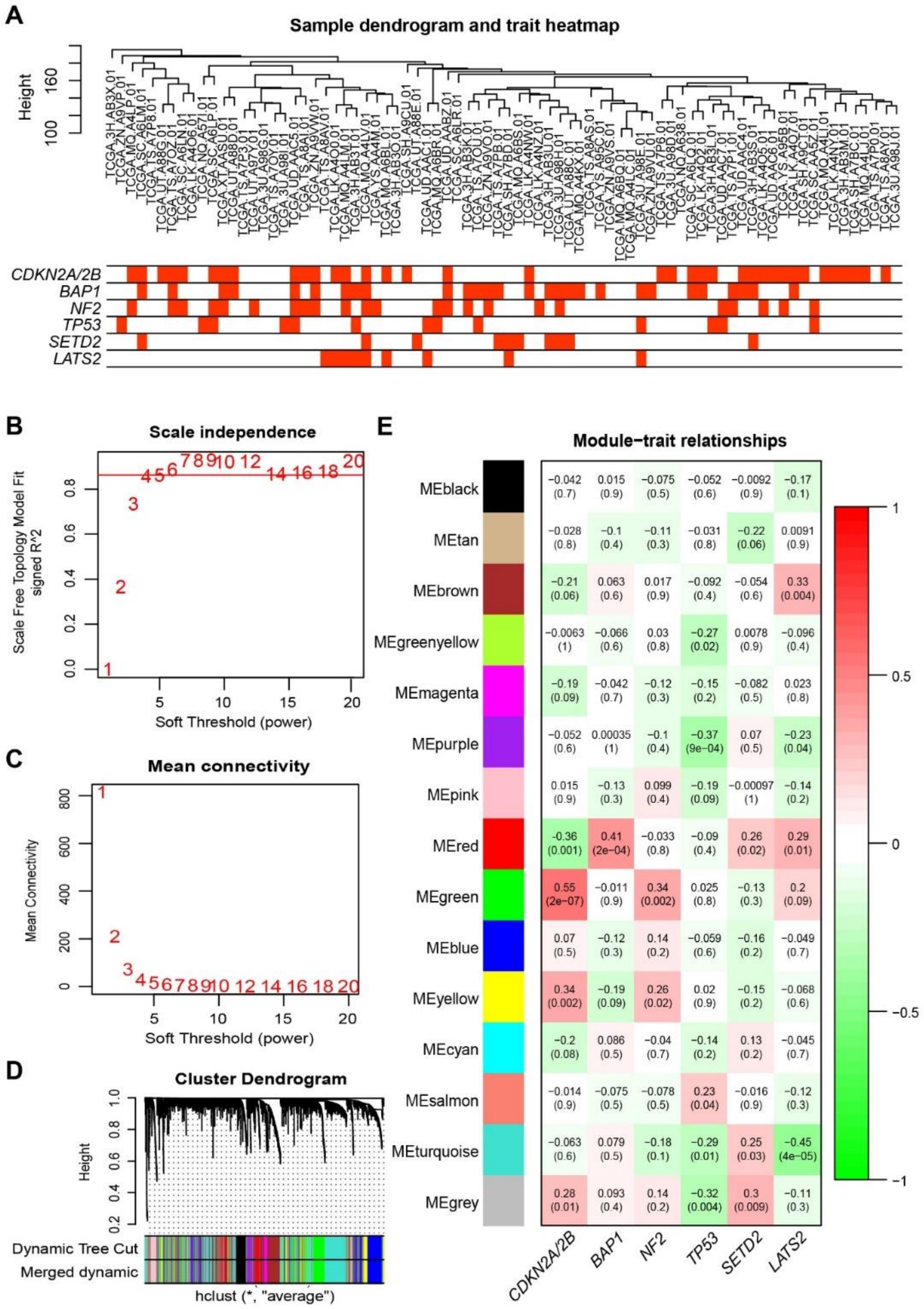

Figure S1. Weighted gene correlation network analysis (WGCNA) reveal gene modules linked with major genetic alterations in MPM. (**A**), Sample dendrogram and trait heatmap across The Cancer Genome Atlas (TCGA) MPM cohort (*N* = 81). (**B**,**C**), WGCNA soft-threshold power determination. Here, we set soft-thresholding power as 5 (scale-free R2 = 0.86), cut height as 0.25, and minimal module size as 30 to identify key modules. (**D**), Gene dendrogram obtained by average linkage

hierarchical clustering. The color row underneath the dendrogram shows the module assignment determined by the Dynamic Tree Cut. Gray genes are unassigned to a module. Gene expression similarity is determined using a pair-wise weighted correlation metric, and clustered according to a topological overlap metric into modules. (**E**), Consensus network modules correlated with major genetic alterations in MPM using the eigenmodule (the first principal component of the module). Correlation coefficient along with *p*-value in parenthesis underneath; color-coded according to correlation coefficient (legend at right). Green color indicates a negative correlation, while red represents a positive correlation.

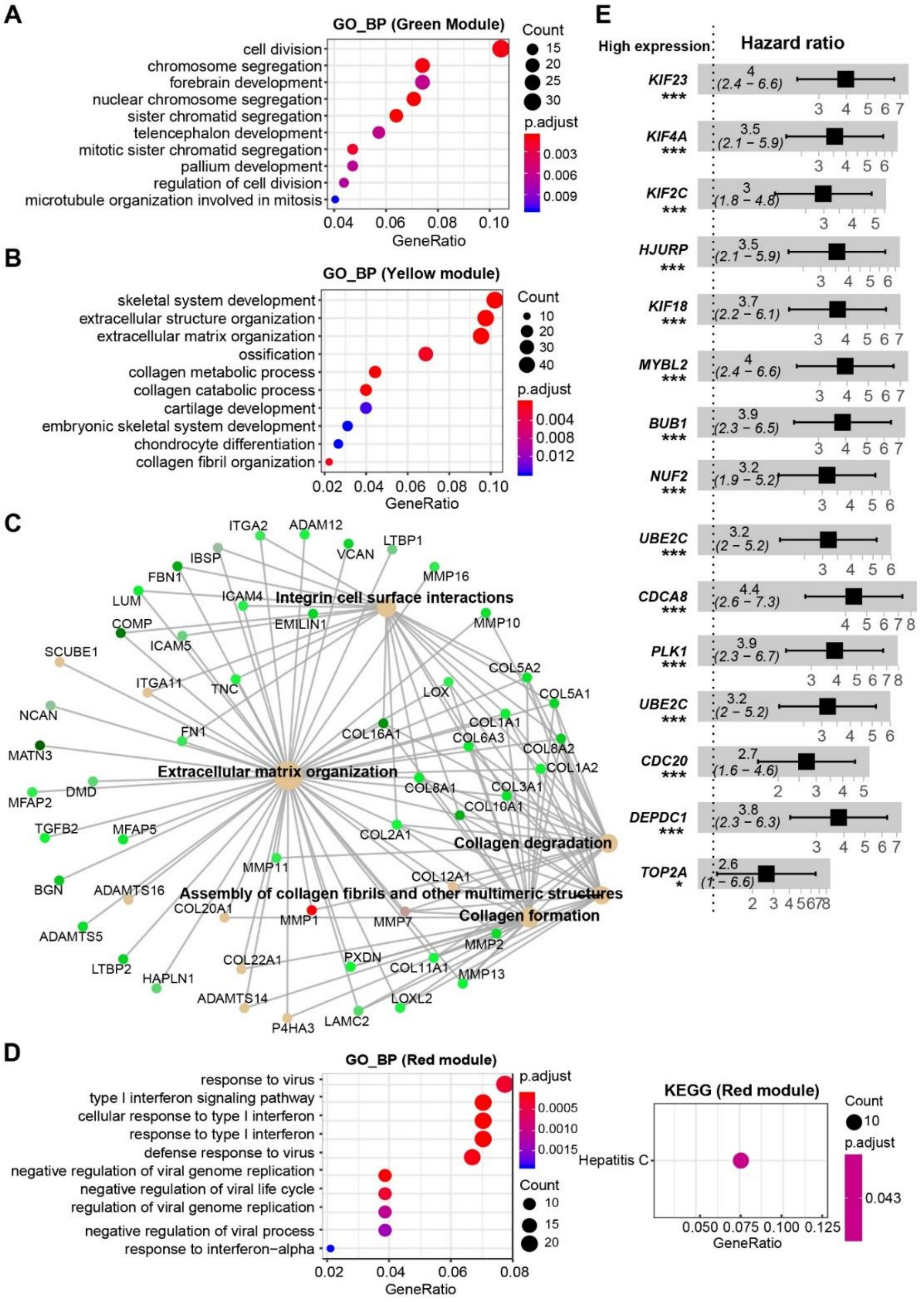

Figure S2. Pathway enrichment analyses of the genes significantly correlated with CDKN2A/2B loss. (**A**–**D**), Top 10 significantly enriched gene ontology (GO) (biological process, BP) pathways based on genes in the green, yellow, red modules. In (**C**), cnetplot was used to list genes in the yellow module based on significantly enriched Reactome pathways. (**E**), Forest plots showed that the top15 connected genes in the green module significantly (*p*-value < 0.05) predicted the overall survival in malignant pleural mesothelioma (MPM) based on The Cancer Genome Atlas (TCGA) MPM cohort.

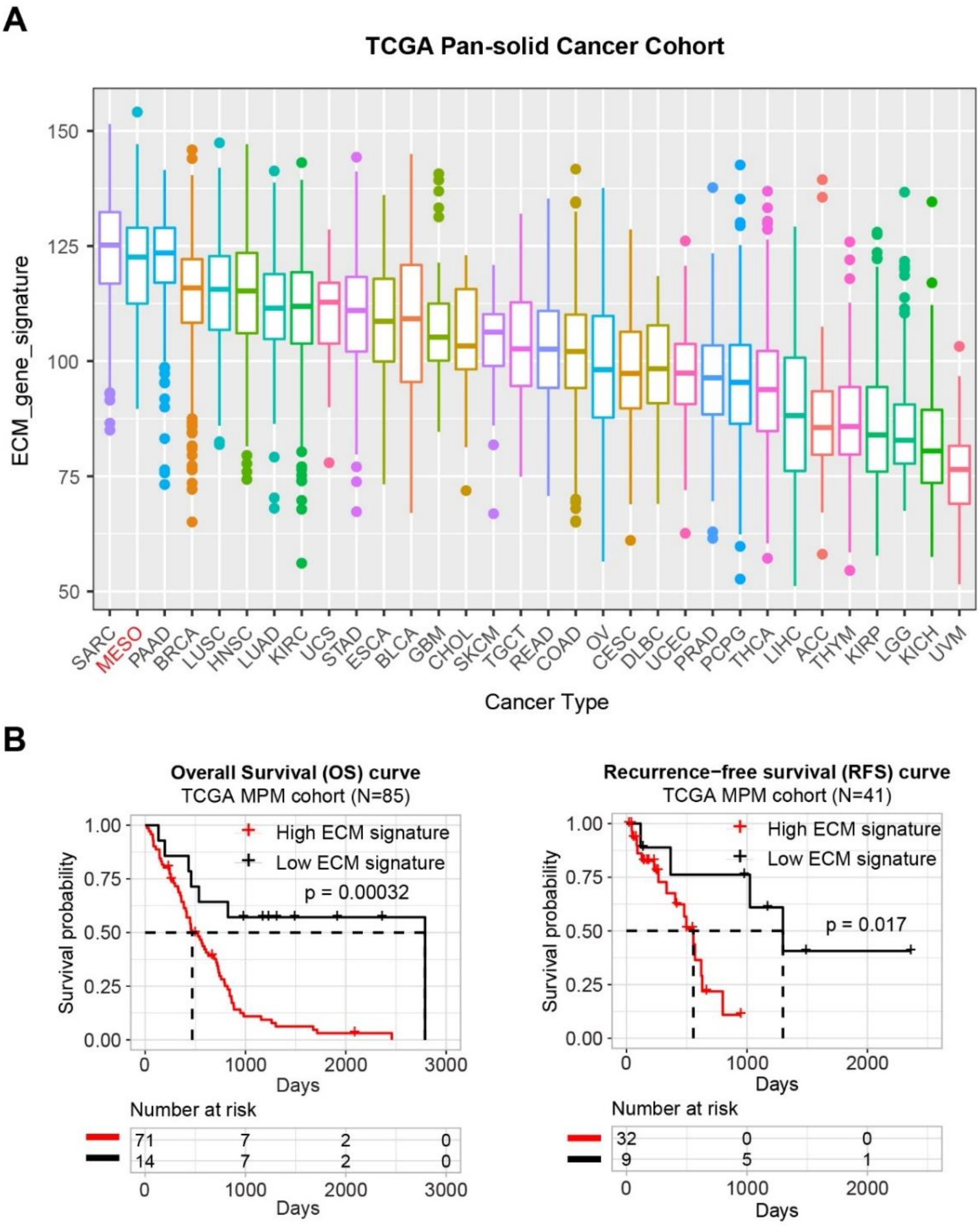

Figure S3. MPM has a high extracellular matrix (ECM) gene signature. (**A**), ECM gene signature across TCGA Pan-solid Cancer Cohort. See the definement of ECM signature in the Methods section. MESO, mesothelioma was highlighted in red. (**B**), Association of ECM signature with OS and DFS in MPM patients. The *p*-value was calculated using the log-rank test.

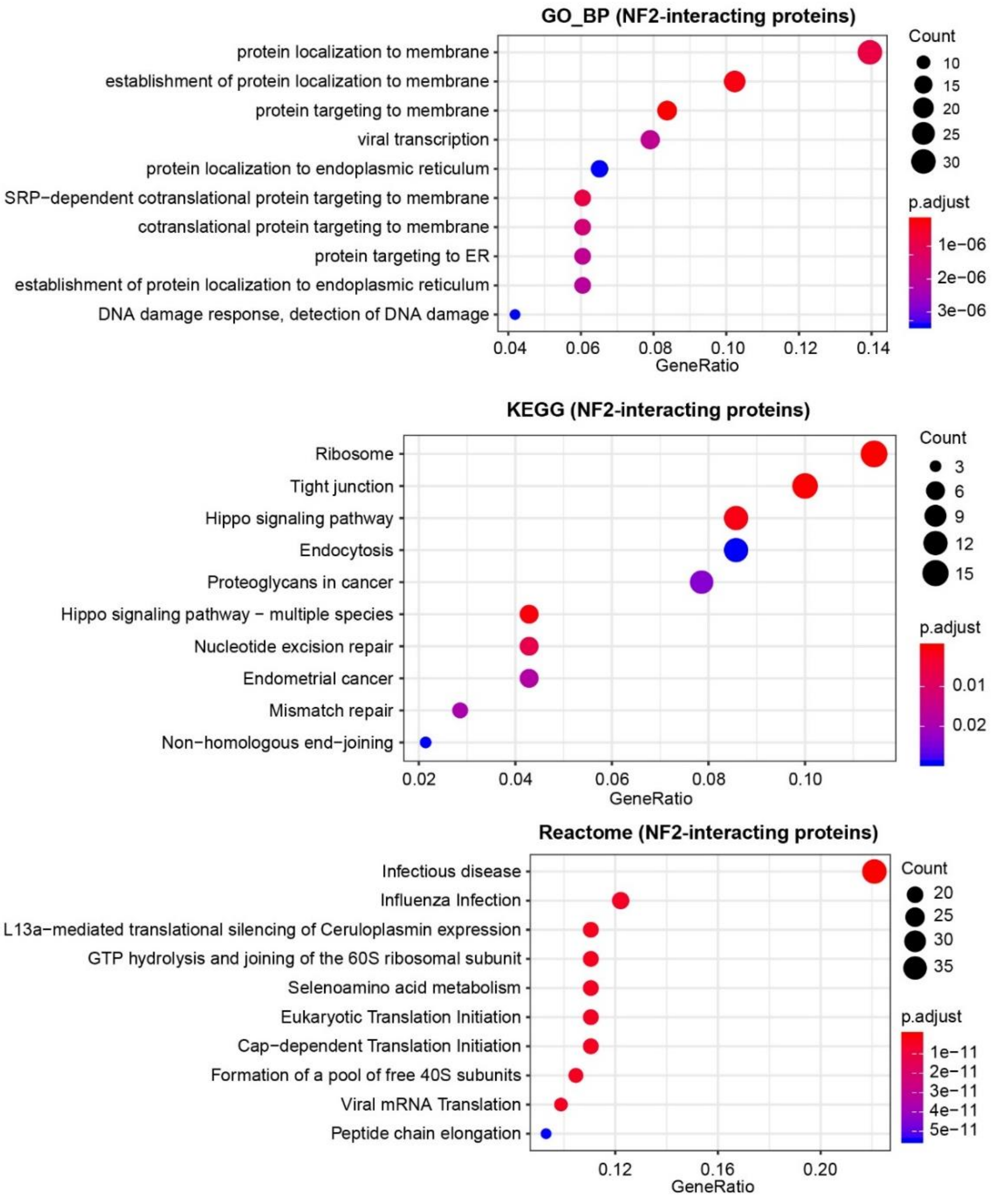

Figure S4. Pathway enrichment analyses of the genes significantly correlated with NF2 alterations (**A**–**D**), Top 10 significantly enriched gene ontology (GO) (biological process, BP), Kyoto Encyclopedia of Genes and Genomes (KEGG) and Reactome pathways based on genes whose encoded proteins physically interact with NF2. Data were downloaded from Agile Protein Interactomes DataServer (http://cicblade.dep.usal.es:8080/APID/init.action).

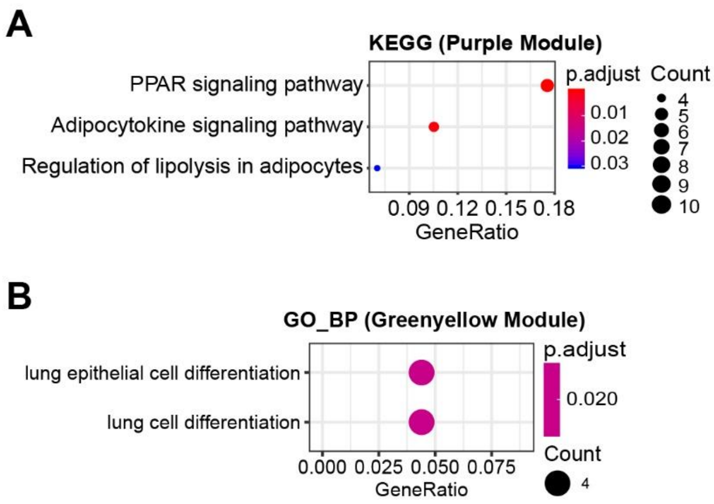

Figure S5. Pathway enrichment analyses of the genes significantly correlated with *TP53* mutations. (**A**,**B**), Significantly enriched Kyoto Encyclopedia of Genes Genomes (KEGG) and gene ontology (GO) (biological process, BP) pathways based on genes enriched in the purple and greenyellow modules.

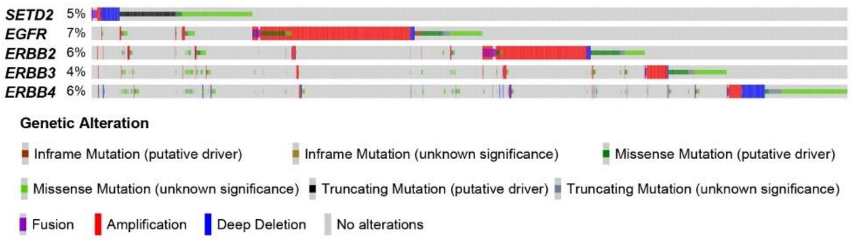

Mutually Exclusive and Co-occurring Analyses

Gene A	Gene B	Neither	A Not B	B Not A	Both	Log2 Odds Ratio	p-Value	q-Value	Tendency
SETD2	*ERBB4*	9184	413	498	94	2.069	<0.001	<0.001	Co-occurrence
SETD2	*ERBB3*	9348	442	334	65	2.041	<0.001	<0.001	Co-occurrence
SETD2	*ERBB2*	9124	435	558	72	1.436	<0.001	<0.001	Co-occurrence
SETD2	*EGFR*	9027	432	655	75	1.259	<0.001	<0.001	Co-occurrence

Figure S6. Mutually exclusive and co-occurring analyses of *STED2* and *EGFR* family genes across TCGA pan-cancer solid tumors. Data of The Cancer Genome Atlas (TCGA) pan-cancer solid tumors were downloaded from cBioPortal (https://www.cbioportal.org/).

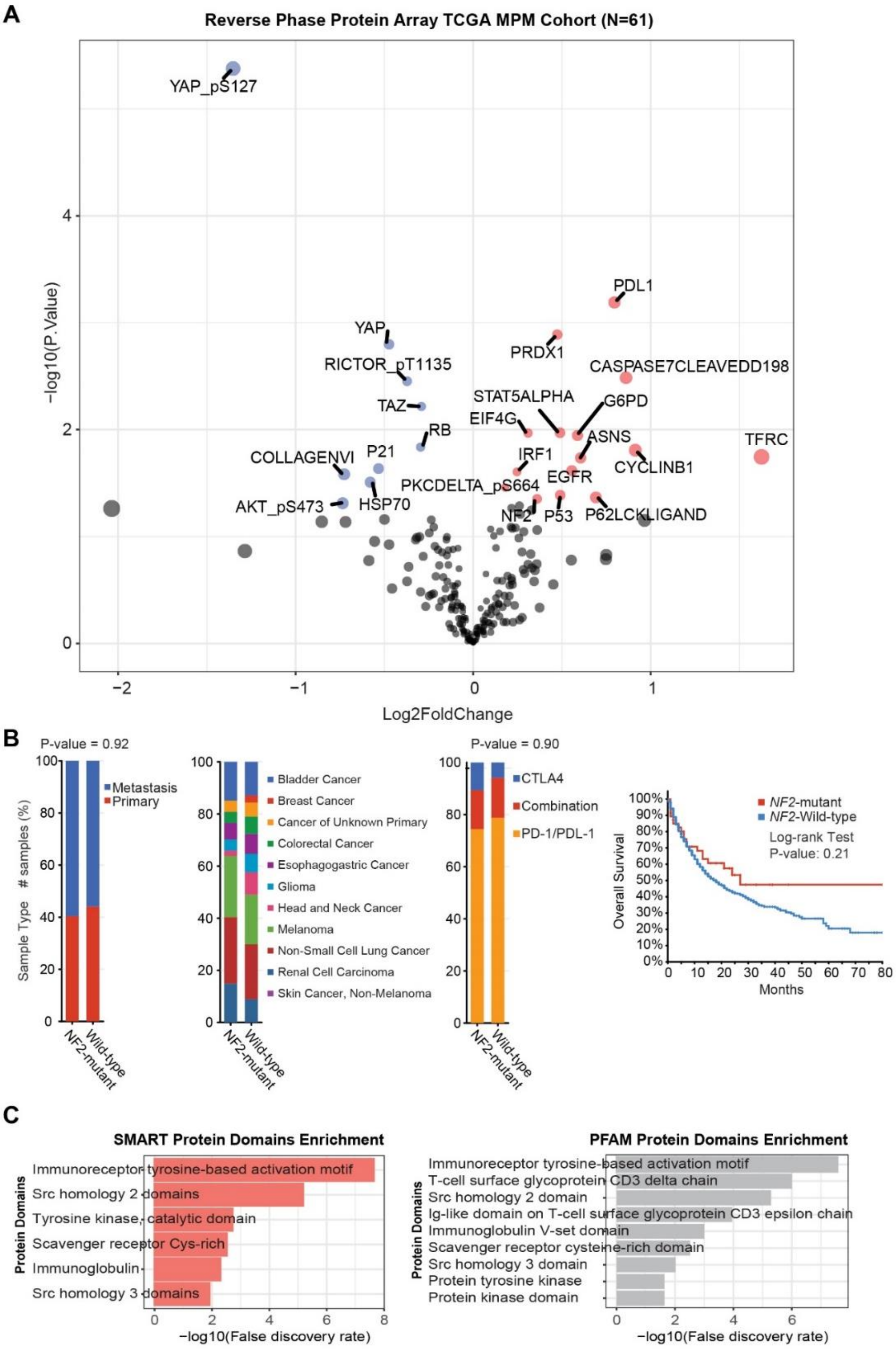

Figure S7. LATS2-altered MPM tumors enrich for the immune-regulatory signature. (**A**), Volcano plot showing the significantly (adjusted *p* value < 0.05) upregulated (red) and downregulated (blue) proteins in malignant pleural mesothelioma (MPM) tumors with LATS2 alterations (versus wild type [WT]) based on The Cancer Genome Atlas (TCGA) MPM cohort (*N* = 61). Data were downloaded and reanalyzed from The Cancer Proteome Atlas (TCPA) database (https://tcpaportal.org/tcpa/). Of note, PDL1 is the highest upregulated proteins in MPM samples with LATS2 alterations. (**B**), NF2 mutations are not associated with significantly improved overall survival in cancer patients after immune checkpoint blockage treatment. The distribution of sample type (primary vs. metastatic; left panel), cancer type (middle panel) and drug type (anti-CTLA4; anti-PD1/PDL1; right panel) between NF2-mutant and wild type cancer. (**C**), Significant (false discovery rate < 0.05) enriched SMART and PFAM protein domains of the top 30 connected genes in the brown module.

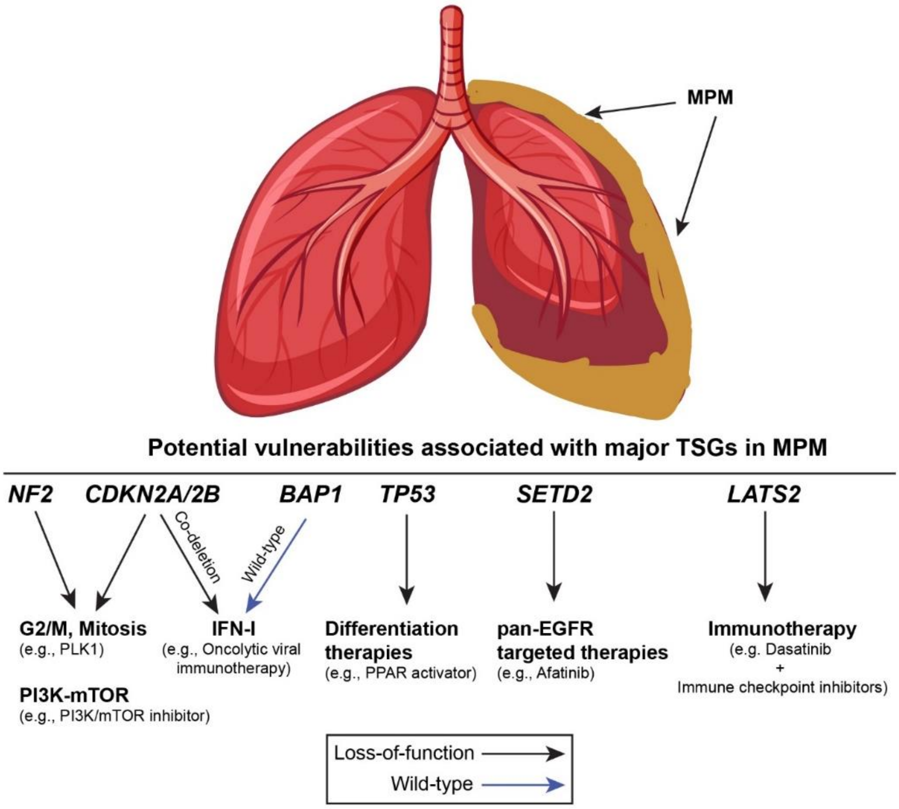

Figure S8. Tumor suppressor genes (TSGs)-guided precision oncology in MPM. A summary of potential targeted- and immunotherapies specified by the TSGs in MPM.

© 2020 by the authors. Licensee MDPI, Basel, Switzerland. This article is an open access article distributed under the terms and conditions of the Creative Commons Attribution (CC BY) license (http://creativecommons.org/licenses/by/4.0/).
